# Association Between Natural Killer Cell Subsets (CD56bright and CD56dim) and Infectious Complications in Newly Diagnosed Multiple Myeloma (NDMM) Patients

**DOI:** 10.3390/cimb48080761

**Published:** 2026-07-26

**Authors:** Ashraf Kakoo, Dara K. Mohammad, Ahmed Yassin, Taban K. Rasheed, Mustafa Al-Attar

**Affiliations:** 1 Biology Department, College of Science, Salahaddin University-Erbil, Erbil 44002, Iraq; ashraf.kako@su.edu.krd (A.K.); taban.rasheed@su.edu.krd (T.R.); mustafa.mustafa@su.edu.krd (M.A.-A.); 2 Center for Hematology and Regenerative Medicine (HERM), Department of Medicine Huddinge, Karolinska Institutet, SE-141 83 Stockholm, Sweden; 3 College of Agricultural Engineering Sciences, Salahaddin University-Erbil, Erbil 44002, Iraq; 4 Department of Internal Medicine, College of Medicine, Hawler Medical University, Erbil 44002, Iraq; ahmed.khudair@hmu.edu.krd

**Keywords:** natural killer cells, CD56bright, CD56dim, infection, multiple myeloma

## Abstract

Background and objective: Natural killer (NK) cells are a subset of CD3^−^CD56^+^ lymphocytes that can recognize and destroy virus-infected and cancerous cells. Infection is the main factor contributing to morbidity and mortality in individuals with multiple myeloma (MM). Therefore, this study was conducted to develop a framework for assessing the association between NK cell subsets (CD56bright and CD56dim) and infections in patients with newly diagnosed multiple myeloma (NDMM). Methods: Peripheral blood samples were obtained from 47 NDMM patients, and NK cell immunophenotype analysis was performed using flow cytometry. Screening for infection was based on the pathogens and combined clinical symptoms. Results: NDMM patients exhibited a significant increase in the overall frequency of circulating NK cells (*p* = 0.001) when compared to healthy controls. Clinically, infectious complications were observed in 31% of the NDMM patients, with viral infections accounting for 19.1% and bacterial infections for 12.7%. Interestingly, phenotypic analysis revealed a marked shift among NK cell subsets, characterized by an expansion of the CD56bright subset (*p* = 0.01) and a concurrent reduction in the CD56dim subset (*p* = 0.01). Additionally, NDMM patients who developed infections had significantly lower NK cell percentages, whereas those without infections showed higher levels, clustering at the upper end. Conclusions: These findings suggest a skewing of the NK cell population toward the CD56bright phenotype. According to the results, NDMM patients should be closely monitored for infection.

## 1. Introduction

Multiple myeloma (MM) is a type of hematologic cancer characterized by the clonal proliferation of malignant plasma cells in the bone marrow [1]. It is often accompanied by a shift in normal immune responses and osteolytic bone diseases. Smoldering MM and monoclonal gammopathy of unknown significance are two asymptomatic precursor diseases that always lead to MM development [2]. The molecular landscape of multiple myeloma is complicated, with significant genetic variation among patients [3]. Many genetic alterations, such as structural genome variants and chromosomal gains and losses, are present in myeloma genomes. These alterations are combined with point mutations that affect different cellular mechanisms, such as genome maintenance. At its worst, MM genome instability manifests as complex genomic rearrangements and mutations [4].

Natural killer (NK) cells are a type of innate lymphocyte that helps control the spread of viral infections and regulates the body’s defenses against tumors; they are essential in the intricate process of cancer growth [5]. These cells, which are specifically associated with hematological malignancies, interact directly with tumor cells, making them appealing targets for cancer treatment [6]. NK cells can eradicate infected or altered cells through a wide variety of inhibitory and activating receptors, as well as adhesion and costimulatory molecules [7]. However, the cytotoxic activity of NK cells against healthy cells is prevented through the interaction between major histocompatibility complex class I (MHC-I) molecules and inhibitory receptors expressed on the NK cell surface. Therefore, the absence or downregulation of MHC-I expression in virus-infected and cancer cells, which prevents the interaction between matching ligands and activating receptors, leads to the killing of the target cell [8].

NK cells are categorized into two subpopulations based on CD56 expression, in which 90% are classed as the CD56dim subset, and 10% are classed as the CD56bright subset [9]. The immature CD56bright subset primarily produces Interferon-γ and TNF-α in response to interleukin-12 and IL-18. On the other hand, the CD56dim subset is thought to be highly cytotoxic and capable of destroying target cells immediately upon interaction with NK receptor ligands [10].

Chimeric antigen receptor (CAR)-NK cells are emerging as a next-generation cellular immunotherapy option, and their favorable safety profile makes them appealing targets for cancer immunotherapy [11]. CAR-NK cells offer several benefits, including the ability to be sourced from allogeneic donors [12]. However, limited knowledge of human NK cell diversity and potential alterations in the tumor microenvironment limit NK cell-based therapy [13].

Infections are a major contributor to morbidity and mortality in patients with myeloma [14]: they were linked to nearly half of all early deaths (less than six months of age) in a study of more than 3000 newly diagnosed multiple myeloma (NDMM) patients. The high susceptibility of MM patients to infection is complex and multifaceted. MM patients have deficiencies in both innate and adaptive immunity, including hypogammaglobulinemia and abnormal immune cell counts, as well as therapy-related changes in immunological status [15].

Despite this, phenotypic assessments of NK cells in NDMM patients and their association with infection are limited; therefore, in this study, we used flow cytometry to investigate NK cell phenotype and disease detection in NDMM patients.

## 2. Materials and Methods

### 2.1. Patients

The Ethics Committee of Salahaddin University’s College of Science in Erbil approved this study (ethics code: 4s/154, date: 10 April 2022). Written informed consent was obtained from all participants for the publication of study data. This study was conducted at Nanakali Hospital for Blood Diseases and Cancer, Erbil, Iraq, between July 2022 and December 2023. None of the patients had received antineoplastic treatment before. Patients were diagnosed with MM according to the International Myeloma Working Group criteria. Peripheral blood samples were collected from 47 NDMM patients and 30 healthy individuals (controls) of the same age. Demographic characteristics of patients were obtained, as were baseline laboratory characteristics related to MM.

### 2.2. Analysis of NK Cell Subpopulation by Flow Cytometry

**Figure 1:**
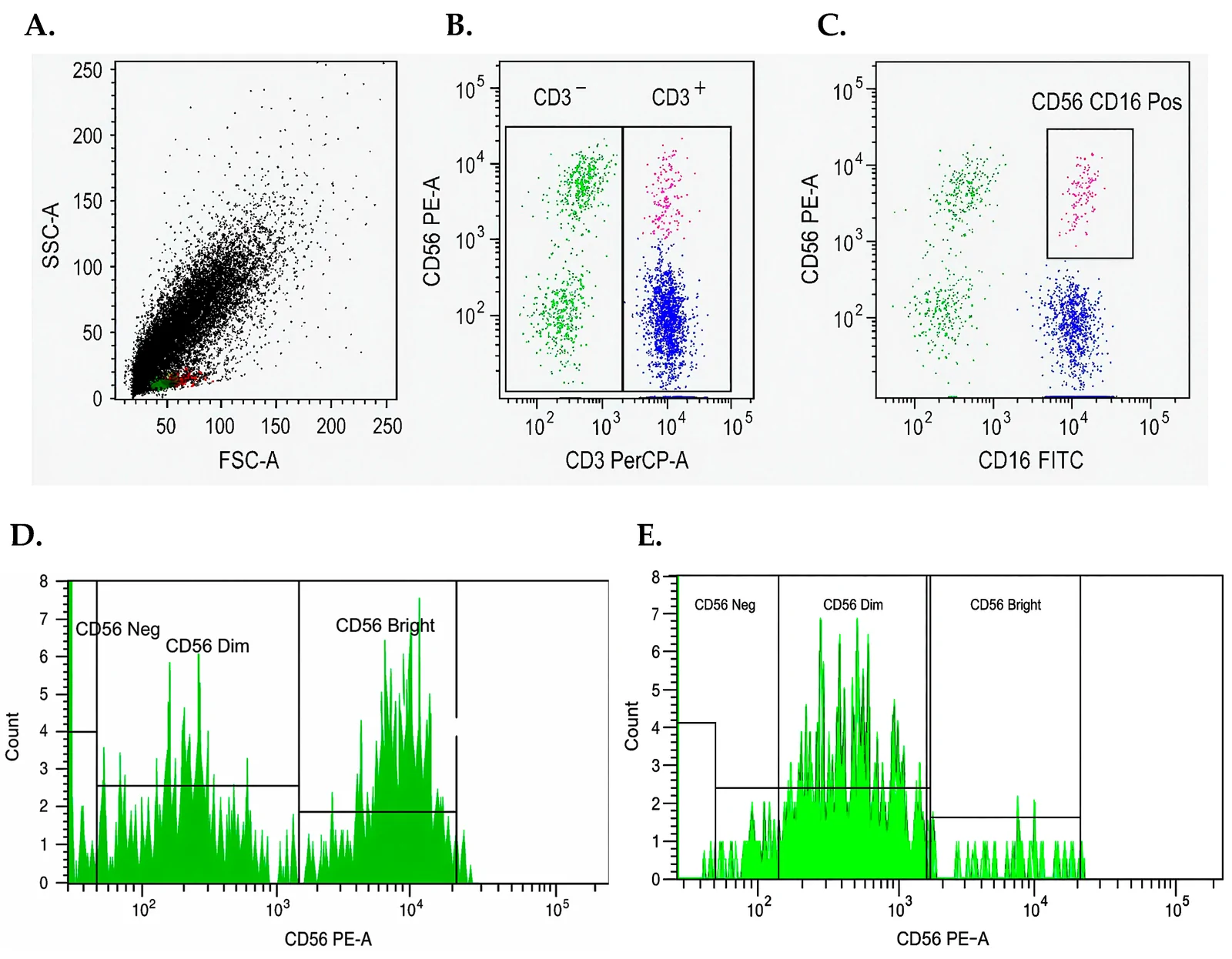
Flow cytometry analysis and sub-gating strategy for human NK cell subsets. (**A**) Forward- and side-scatter plot used to gate total viable lymphocyte populations. (**B**) CD3 vs. CD56 plot showing exclusion of CD3^+^ T cells and selection of CD3^−^CD56^+^ NK cells. (**C**) CD16 vs. CD56 distribution of NK cells, with the boxed region indicating the CD56^+^CD16^+^ subset. (**D**,**E**) Histogram of CD56 expression illustrating separation of CD56^−^, CD56dim, and CD56bright NK cell populations in NDMM patients (**D**) and healthy controls (**E**). NDMM patients (*n* = 47) and healthy individuals (*n* = 30).

Immunophenotype analysis was performed using a FACS Canto (Becton, Dickinson, San Jose, CA, USA) flow cytometer, and the data were collected using the FACS Diva program (Figure 1). Blood samples were collected in EDTA tubes from NDMM patients and healthy individuals to study NK cell phenotype. First, the following fluorescence-labeled conjugated monoclonal antibodies were used to identify the cells in the samples: CD3 PerCP (Peridinin–Chlorophyll–Protein)/CD56 PE (Phycoerythrin) and CD16 FITC (Fluorescein isothiocyanate) (all from Becton Dickinson, USA). After that, the cells mixed with antibodies were incubated in the dark at room temperature for 30 min. Fluorochrome-conjugated isotype-matched antibodies were used as negative controls. Then, the “Ammonium Chloride-containing Lysing Solution” was used to lyse the erythrocytes; afterward, the prepped cells were placed in the flow cytometer’s laser beam.

### 2.3. Detection of Infections

Infections were diagnosed based on the identification of a pathogen, radiological imaging (X-ray and CT scan), and concurrent clinical symptoms, such as fever not attributable to medication, a productive cough, dysuria, and so on. After culturing the bacteria, VITEK was used to identify them. Epstein–Barr virus (EBV), Herpes Zoster virus, and influenza virus were the most common viruses identified by polymerase chain reaction (PCR), a technique that amplifies DNA or RNA. Infection was assessed at the time of diagnosis, before treatment initiation.

### 2.4. Statistical Analysis

Version 6.0 of GraphPad Prism was used to create the graphics and perform statistical analyses. The data were examined using the D’Agostino–Pearson omnibus test, the Shapiro–Wilk normality test, and the Kolmogorov–Smirnov test to assess normality. To compare values between patients and the control group, a *t*-test was used for normally distributed data, with results reported as means ± SE (standard error); if the data were not normally distributed, the Wilcoxon test was used, with results reported as median (range). The Kruskal–Wallis test was used to compare groups. For every analysis, *p* values less than 0.05 were deemed statistically significant.

## 3. Results

### 3.1. Patient Characteristics

This study included 47 NDMM patients with a mean age of 67 years. Males represented 61.7% of the cohort. IgG myeloma was the most common isotype (53.2%), followed by IgA (23.4%), light chain multiple myeloma (14.9%), and non-secretory multiple myeloma (8.5%). According to the International Staging System (ISS), 48.9% of the patients were classified as stage III. Laboratory findings showed elevated median creatinine and urea levels, reduced albumin and hemoglobin levels, and increased β2-microglobulin, erythrocyte sedimentation rate (ESR), and C-reactive protein (CRP) levels, reflecting significant disease burden and systemic inflammation (Table 1).

### 3.2. Natural Killer Cell Phenotype

**Figure 2:**
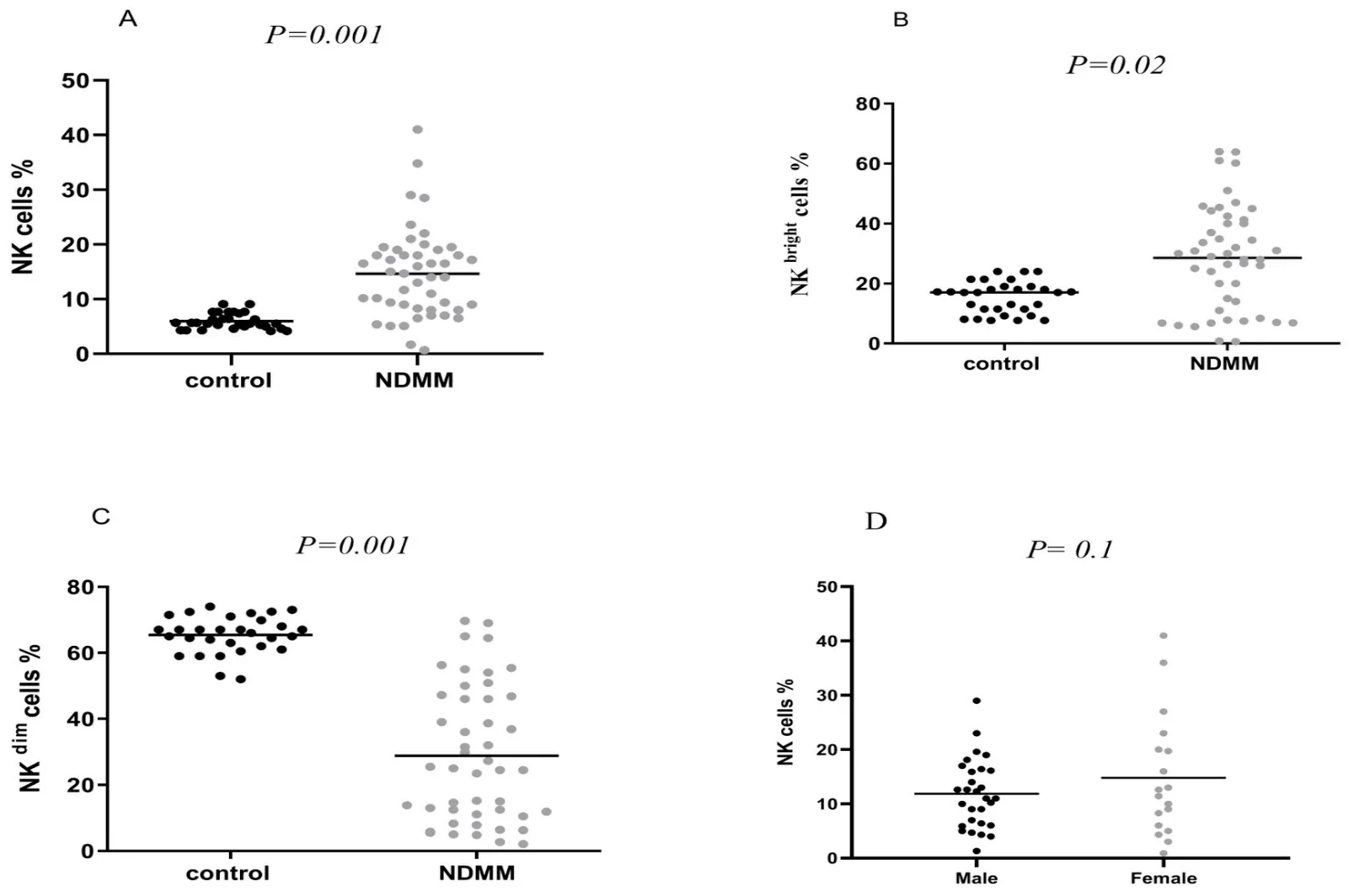
Alteration of natural killer cell phenotype in newly diagnosed multiple myeloma (NDMM) patients. (**A**) Total NK cell frequencies (CD3^−^CD56^+^) are significantly higher in NDMM patients than in healthy controls (*p* = 0.001). (**B**) The proportion of CD56bright cells is elevated in NDMM patients compared with controls (*p* = 0.02). (**C**) The percentage of CD56dim cells is markedly reduced in NDMM patients relative to controls (*p* = 0.001). Data are presented as a scatter dot plot showing group distributions. These findings indicate a shift toward a more immature NK cell phenotype in NDMM. (**D**) The percentage of circulating NK cells in male and female patients with NDMM. NDMM patients (*n* = 47) and healthy individuals (*n* = 30).

The phenotypic characteristics of circulating NK cells revealed a noticeable alteration of the NK cell distribution in the patients with NDMM, who had a significantly higher median percentage of total NK cells (13.5%) than the healthy controls (5.6%) (*p* = 0.001), indicating an overall expansion of the NK cell population (Figure 2A). Further subset analysis revealed a distinct shift toward the immunomodulatory CD56bright subset. Specifically, the NDMM patients showed a substantial increase in the median proportion of CD56bright cells (28.3%) compared with healthy individuals (17%; *p* = 0.02; Figure 2B). In contrast, the cytotoxic CD56dim subset, which generally constitutes the majority of NK cells in peripheral blood, was significantly decreased in NDMM patients. The median percentage of CD56dim cells was reduced from 65.0% in the healthy controls to 27.1% in the NDMM patients (*p* = 0.001) (Figure 2C). Collectively, these findings highlight a pronounced redistribution of NK cell subsets in NDMM, characterized by the expansion of the CD56bright compartment and a concomitant contraction of the CD56dim cell population (Figure 2).

**Figure 3:**
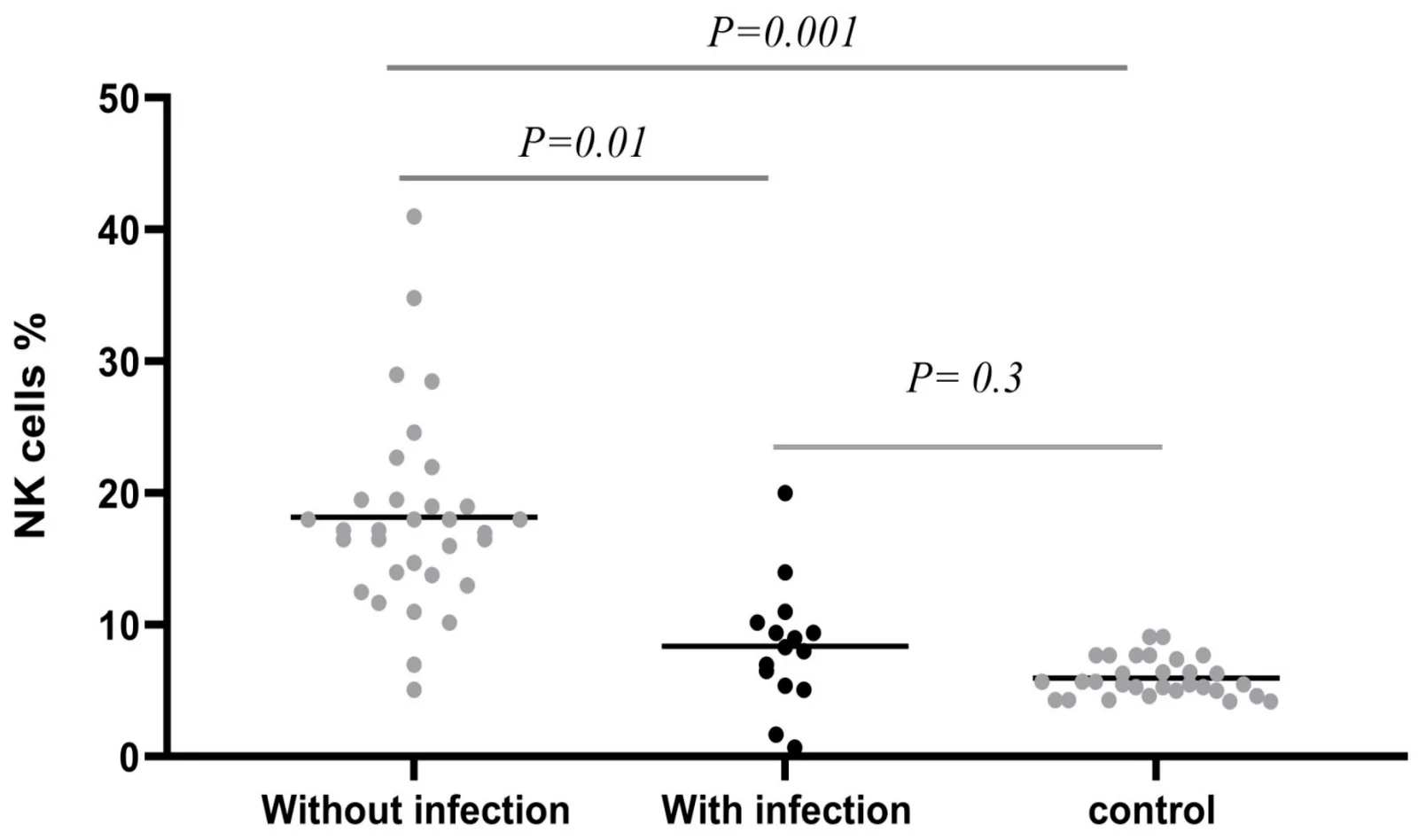
Total natural killer cell percentages in NDMM patients with and without infection compared to healthy controls. Scatter dot plots illustrate the distribution of circulating NK cells (% NK cells) among NDMM patients who remained infection-free, those who developed infectious complications, and healthy controls. NDMM patients with infection (*n* = 15), without infection (*n* = 32) and healthy individuals (*n* = 30).

Next, we compared the NK cell distribution between the NDMM patients with and without infectious complications and found a significant decrease (*p* = 0.01) in the total NK cell percentage among those who developed infections (Figure 3). Additionally, the NDMM patients without infection exhibited higher NK cell levels, with values clustering around the upper range of the cohort (Figure 3). These findings indicate that diminished NK cell abundance may be associated with increased susceptibility to infection in NDMM, suggesting that impaired NK cell homeostasis could contribute to compromised immune defense early in the disease’s course.

**Figure 4:**
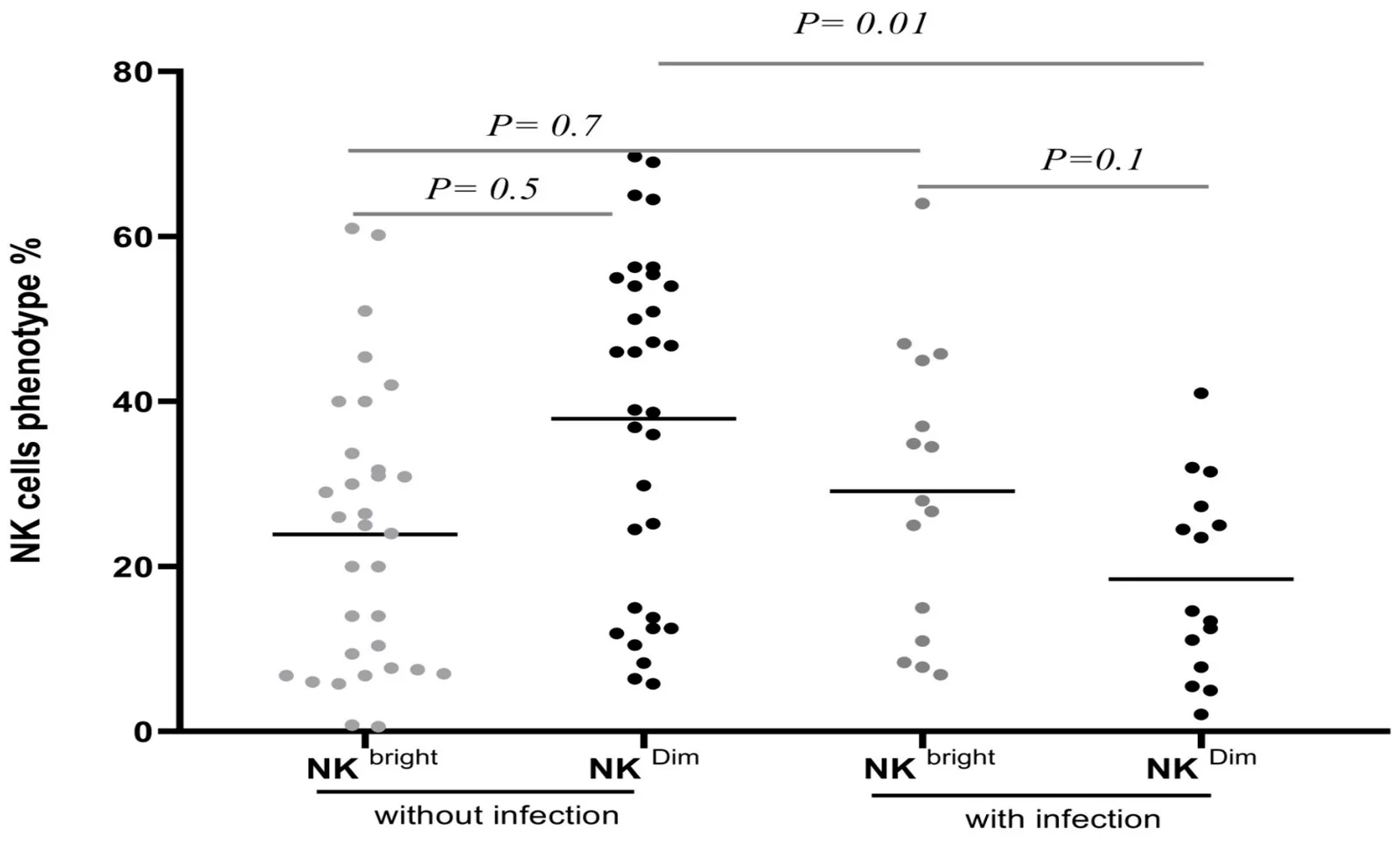
Natural killer cell phenotypes in the NDMM patients with and without infection. Bar graph showing the percentage of NK cells exhibiting bright (high CD56 expression) or dim (low CD56 expression) phenotypes in NDMM patients, grouped by the presence or absence of infection. Data are presented as mean percentages, with *p* values indicating significant differences between groups. CD56dim cells were significantly reduced in infected patients (*p* = 0.01) compared to non-infected patients, whereas CD56bright cells did not differ significantly between groups (*p* = 0.7). NDMM patients with infection (*n* = 15) and without infection (*n* = 32).

Moreover, an analysis of the natural killer (NK) cell subset distribution revealed a significant shift in NK cell phenotype in NDMM patients with active infection compared to those without active infection. Specifically, the CD56dim cytotoxic subset was markedly less frequent in infected patients (median 16%) than in non-infected patients (median 46%; *p* = 0.01) (Figure 4). In contrast, the immunomodulatory CD56bright subset showed a non-significant numerical increase in the infected group (median 35%) compared to the non-infected group (median 31.5%; *p* = 0.7). Consequently, the relative balance between these subsets was altered within the infected and non-infected cohorts. The proportion of CD56bright cells was not significantly different from CD56dim cells (*p* = 0.1), whereas in the absence of infection, CD56dim cells constituted a larger fraction of the NK compartment than CD56bright cells (*p* = 0.5). These data indicated that infection in NDMM patients is associated with a significant reduction in the cytotoxic CD56dim pool and a shift in the overall NK subset ratio (Figure 4).

### 3.3. Incidence of Infection in NDMM Patients

**Figure 5:**
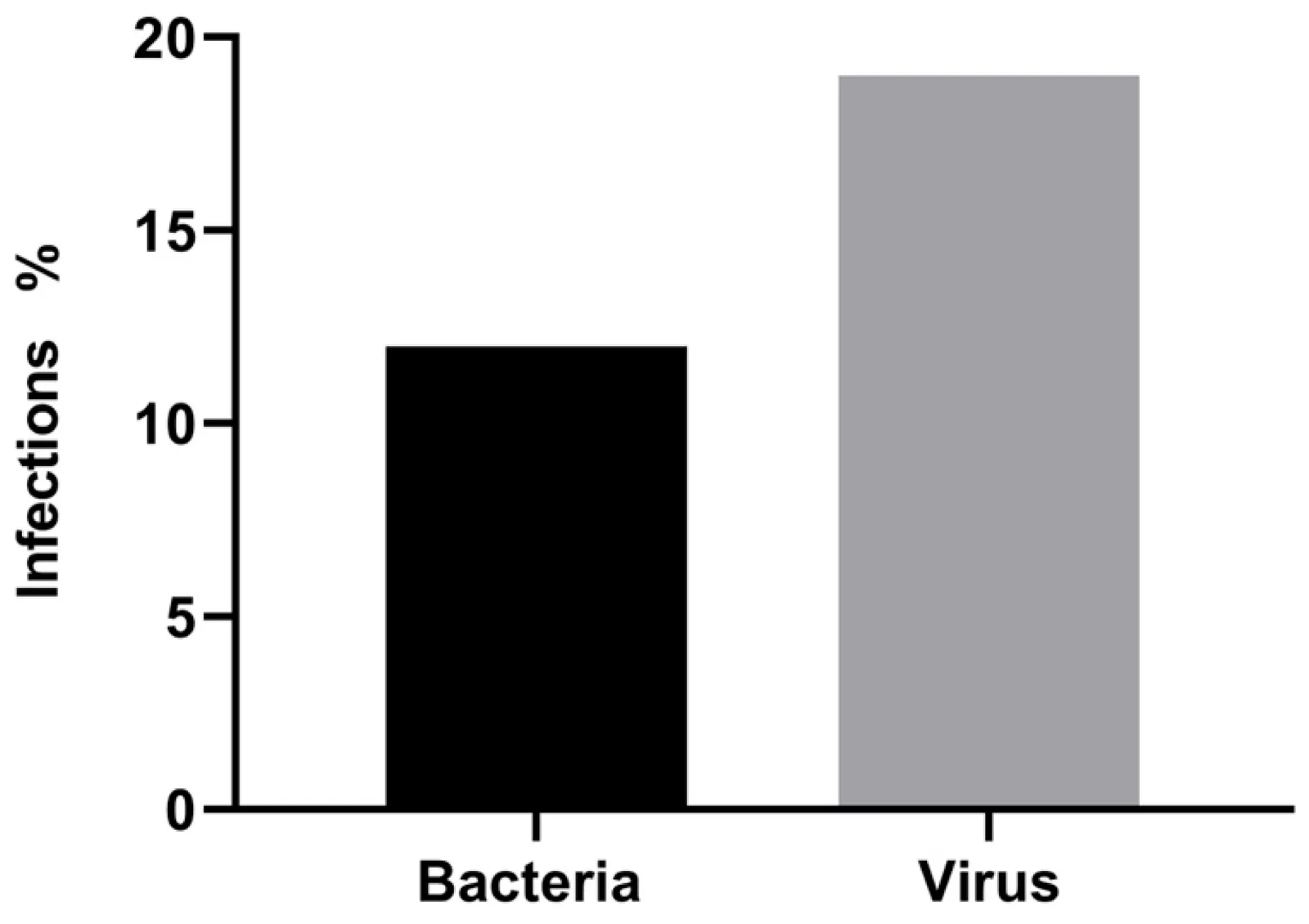
Types of infections in NDMM patients. Bar chart illustrating the proportion of total documented infections in the study cohort attributed to bacterial (black) versus viral (gray) pathogens. The relative size of each segment represents the percentage of infections caused by each category. NDMM patients (*n* = 47) and healthy individuals (*n* = 30).

Out of all the patients, 31.8% exhibited infection, of which viral infections (19.1%) were the leading cause, including Epstein–Barr virus (EBV), Herpes Zoster virus, and influenza virus. The second-highest cause of disease was bacteria, at 12.7%, including *Streptococcus pneumoniae*, *pulmonary tuberculosis*, *Staphylococcus aureus*, and *Escherichia coli* (Figure 5 and Table 2). No patients suffered from fungal infections.

**Figure 6:**
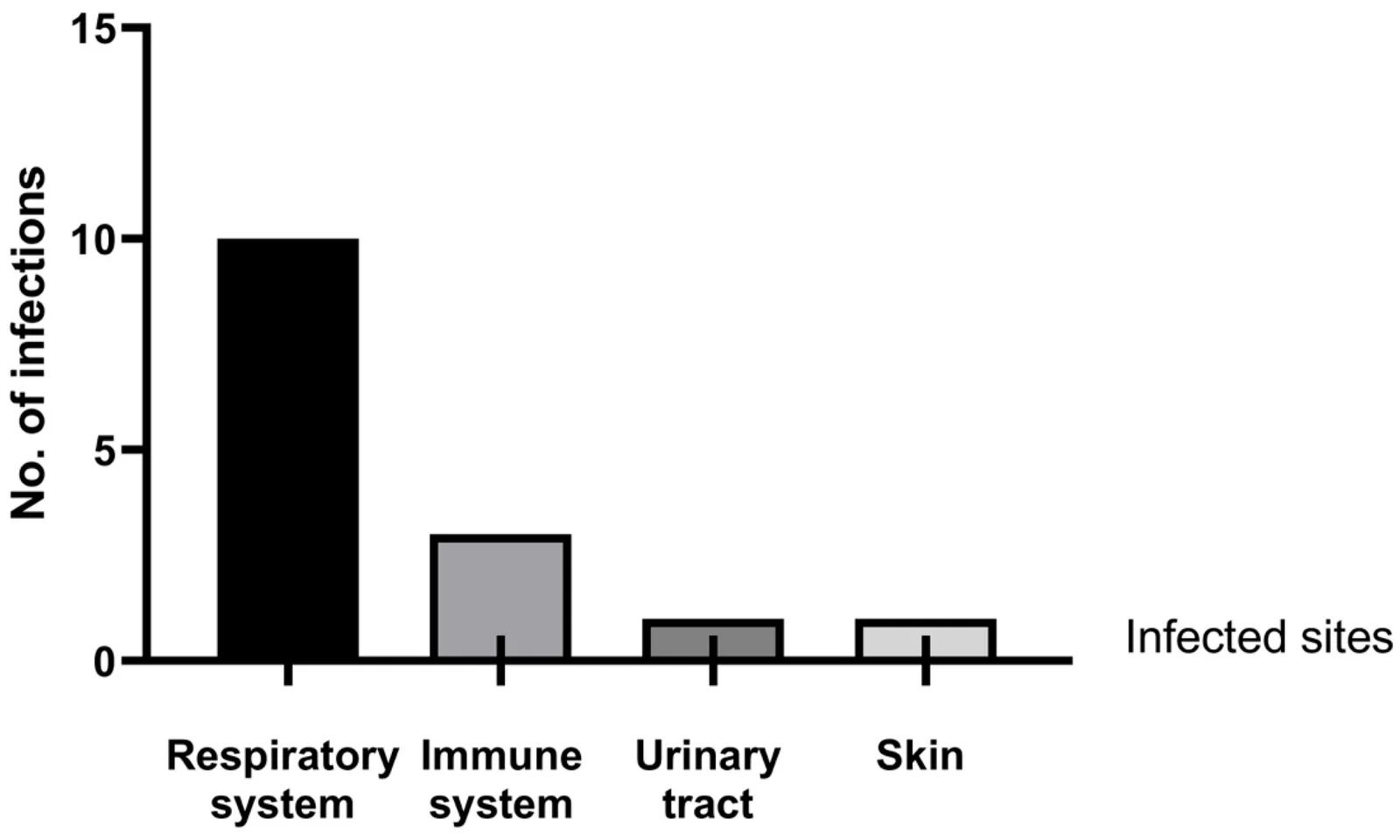
Distribution and frequency of infection sites in NDMM patients. Bar graph depicting the total number of recorded infections categorized by primary anatomical site: respiratory system, immune system (e.g., disseminated viral), urinary tract, and skin. The height of each bar corresponds to the incidence count. Common causative pathogens are annotated for each site: respiratory infections involved viral (Epstein–Barr virus, influenza virus) and bacterial (*Streptococcus pneumoniae*, *Mycobacterium tuberculosis*) agents; immune system infections were primarily due to Herpes Zoster virus reactivation; *Escherichia coli* most frequently caused urinary tract infections; and skin infections were commonly caused by *Staphylococcus aureus* (cellulitis). NDMM patients (*n* = 47) and healthy individuals (*n* = 30).

Additionally, the distribution of infection sites among patients revealed a distinct anatomical pattern. As shown in Figure 6, the respiratory system was the most frequent site of infection, accounting for the highest number of documented cases. This was followed sequentially by infections of the immune system (primarily manifesting as viral reactivations), the urinary tract, and the skin. Microbiological analysis was performed to identify the causative agents at each major site: Respiratory infections were polymicrobial, involving pathogens such as *Streptococcus pneumoniae*, *Mycobacterium tuberculosis*, Epstein–Barr virus, and influenza virus. Immune system infections were predominantly driven by reactivation of Varicella–Zoster virus (Herpes Zoster). Urinary tract infections were caused by *Escherichia coli*, while skin infections, primarily cellulitis, were caused by *Staphylococcus aureus*. This stratification underscores the respiratory tract as the primary focus of infectious morbidity in this cohort, with specific etiological agents characterizing each site of infection (Figure 6).

**Figure 7:**
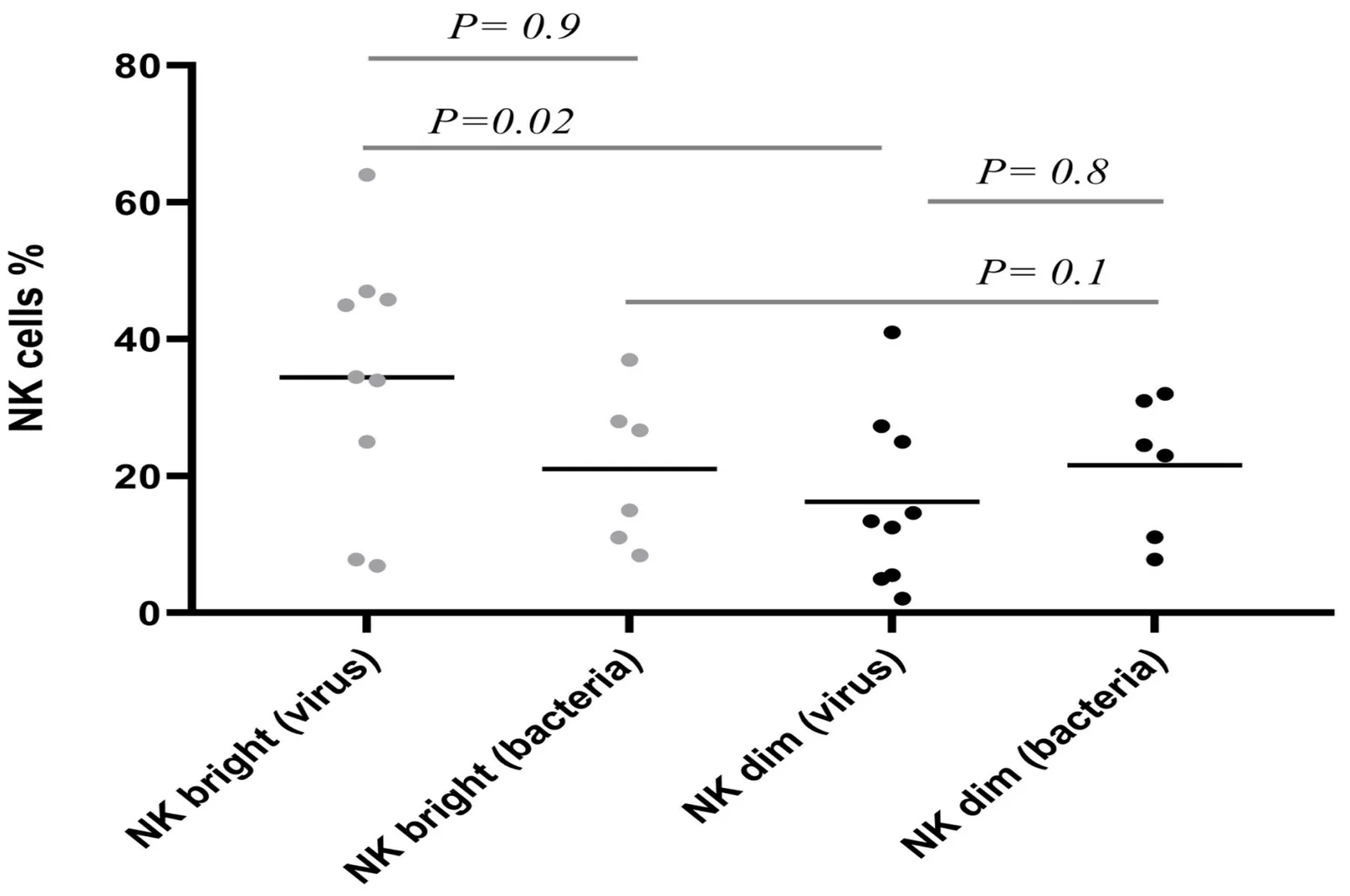
Natural killer cell phenotype in patients with bacterial and viral infections. Box-and-whisker plots illustrating the percentage of NK cell subsets, including CD56bright and CD56dim populations, in viral and bacterial conditions. Statistical comparisons between groups are indicated above the plots: CD56bright (virus) vs. CD56bright (bacteria) (*p* = 0.9), CD56bright (virus) vs. CD56dim (virus) (*p* = 0.02), CD56dim (virus) vs. CD56dim (bacteria) (*p* = 0.8), and CD56bright (bacteria) vs. CD56dim (bacteria) (*p* = 0.1). These comparisons highlight a significant difference between the CD56bright (virus) and CD56dim (virus), while other comparisons do not reach statistical significance. NDMM patients (*n* = 47) and healthy individuals (*n* = 30).

Moreover, we compared circulating NK cell subsets between patients with viral and bacterial infections (Figure 7). The distribution of natural killer (NK) cell subsets differed modestly between viral and bacterial conditions, with greater variability observed within the CD56bright compartment than the CD56dim compartment. As shown in the box-and-whisker plots (Figure 7), the proportion of CD56bright cells was higher in the viral group compared with the bacterial group, although this difference did not reach strong statistical significance overall; a pairwise comparison between the CD56bright (virus) and CD56dim (virus) groups indicated a modest but statistically significant difference (*p* = 0.02). In contrast, CD56dim frequencies were lower and more variable in the viral condition than in the bacterial condition, with no statistically significant difference between CD56dim (virus) and CD56dim (bacteria) (*p* = 0.8). Overall, these findings suggest that infection type may exert a limited, subset-specific influence on NK cell distribution, with the most notable effect observed in the CD56bright population.

## 4. Discussion

Immunological cells, such as NK cells, are essential for regulating tumor development and the spread of intracellular infections. As observed in the present study, there was a range in the percentages of CD56bright NK cells and CD56dim NK cells in NDMM. In this investigation, NDMM patients showed a significantly higher frequency of NK cells than the control group. When comparing the NDMM to the control, a notable skew toward the CD56bright population and a decline in the CD56dim population were observed. Previous studies have demonstrated alterations in NK cell phenotype and function in NDMM using comprehensive phenotypic and functional analyses [10]. Our findings are consistent with these observations; however, our study evaluated a more limited panel of NK cell markers and did not include functional assays.

There have been reports of decreased CD56dim NK cells in various cancers, such as non-small cell lung cancer [10] and bladder cancer [16]. In hematologic malignancies, a study found that the blood NK cells of patients with Hodgkin lymphoma were enriched in the CD56bright fraction [17]. In contrast, another study found that the CD94lowCD56dim subgroup was more abundant in myeloma patients. Changes in the distribution of NK cell subsets are correlated with tumor-induced impairment of NK cell activity [8]. The mechanism underlying changes in the NK cell phenotype in MM patients remains unknown. A probable explanation is that myeloma-related substances could be released into the bloodstream, affecting NK cells systemically. MM is characterized by a robust inflammatory network [18], and many cytokines observed at high concentrations in the serum of myeloma patients could influence NK cell transcriptomic changes. These include NFκB signaling and cytokines such as TNF and IL-1β, which are associated with inflammation [19].

Additionally, a cluster of NK cells associated with MM were identified through the expression of interferon-stimulated genes. These genes commonly include IFI44L, ISG15, MX1, IFIT1, IFIT3, and OAS1. The expression of these genes suggests chronic exposure to interferon signaling within the MM microenvironment, potentially driven by persistent immune activation, tumor-derived signals, or therapy-induced immune modulation [20]. This suggests that NK cells in MM may be affected by other inflammatory factors associated with MM, such as type I interferons [21]. It is worth noting that several of the previously described factors contribute to the growth of immunosuppressive cells in the MM microenvironment, including mesenchymal stem cells (MSCs) and T regulatory cells [22], which may directly reduce NK cell activity. There are many new therapies, such as proteasome inhibitors (PIs) and Immunomodulatory Drugs (IMiDs), that affect NK cell function and may help explain how NK cells influence therapy outcomes. For example, it has been demonstrated that PI increases NK cell activity in cancer patients [23].

Patients with MM typically have immunological paresis and different levels of immune weakness, which increases the chance of developing severe infections [24]. This study’s data shows that 31% of the NDMM patients experienced infections, and the percentages of total NK cells and CD56dim NK cells in the infected patients were significantly lower than in non-infected individuals. Changes in NK cell phenotype may increase susceptibility to infections in MM patients [25]. Defects in CD56dim lead to reduced proliferation, decreased NK cell activation, reduced IFNγ production, and increased PD1 expression [10]. Patients with MM who are newly diagnosed have varying survival times, which can range from a few days to over ten years [15]. A previous study attested to the fact that infections pose a serious risk to MM patients; the survival of MM patients with infections was considerably lower than that of patients without infections [26].

The main pathogen causing infection in this study was viral, and the respiratory system was the most common site of infection, which agrees with many previous studies [15,27]. Some viral infections, particularly Epstein–Barr virus and Herpes Zoster, may represent viral reactivation rather than primary infection. According to one study, people with multiple myeloma were at a high risk of having any infection; the chance of a bacterial infection was seven times higher, and the risk of a viral infection was ten times higher [28]. Additionally, prior research has shown that patients with MM are more likely to have Epstein–Barr virus infections [15]. Gram-negative bacteria, on the other hand, are the most common pathogens in NDMM patients, according to numerous published studies [29,30]. Therefore, our results align with the existing evidence and further strengthen our current understanding of NK cell dysregulation in disease contexts.

Although this study showed an increase in the total number of NK cells, the patients exhibited an unusual NK cell phenotype; moreover, there was a significant decline in the total and CD56dim NK cell counts in patients with infection. A limitation of the present study is the lack of functional validation of NK cell activity. While we characterized the distribution of NK cell subsets (CD56bright and CD56dim), we did not assess their cytotoxic capacity or effector molecule expression, such as through intracellular quantification of perforin and granzyme content, due to resource, time, and platform constraints. As a result, our findings remain descriptive and do not establish whether the observed differences in NK cell subset distribution translate into altered functional activity. Future studies incorporating standardized cytotoxicity assays and effector molecule profiling will be essential to determine the biological and clinical relevance of these phenotypic differences. Another limitation is that the relatively small sample size may have restricted the statistical power to detect subtle differences among patient subgroups. The variations in disease burden, including ISS stage, renal function, albumin levels, and inflammatory markers, may have influenced both infection risk and NK cell subset distribution. The pathogen-specific analyses are descriptive and exploratory due to the small numbers within individual infection categories. Accordingly, the primary inferential analysis focuses on comparing patients with any infection and those without infection, whereas detailed pathogen-specific findings are presented only as supportive observations. However, multiple myeloma is a relatively uncommon hematological malignancy, and the number of newly diagnosed patients available during the study period was limited. Therefore, only eligible NDMM patients who met the inclusion criteria and presented to our center during the study period were enrolled. An additional limitation of this study is that NK cell subsets were evaluated as relative percentages rather than absolute cell counts. Although relative frequencies provide valuable information regarding the distribution of immune cell populations, they do not reflect the actual number of circulating NK cells. Absolute counts may more accurately represent the degree of immune competence, particularly in patients with hematological malignancies, where fluctuations in total lymphocyte numbers are common. Consequently, incorporating absolute NK cell counts in future studies may improve the assessment of immune dysfunction and strengthen the evaluation of associations between NK cell abnormalities and infection risk. Additionally, the cross-sectional design did not allow for the assessment of temporal changes in NK cell subsets, and no causal relationships could be established. Moreover, the study investigated only circulating NK cell populations and did not evaluate NK cells in the bone marrow microenvironment, a crucial site for multiple myeloma development and immune regulation. Potential confounding factors include disease heterogeneity, treatment-related variables, and infection-associated immunomodulation that may have affected the NK cell phenotypes. These findings need to be validated and extended in future studies with larger cohorts, longitudinal monitoring, and comprehensive functional assessments.

## 5. Conclusions

NDMM patients exhibited a distinct alteration in circulating NK cell subset distribution, characterized by an increased proportion of CD56bright cells and a decreased proportion of CD56 dim cells compared with healthy controls. Infectious complications were common in this study, and patients with infections showed lower NK cell percentages than those without infections. These findings suggest an association between NK cell subset imbalance and infection status in NDMM; however, larger studies incorporating functional NK cell analyses are required to clarify the biological and clinical significance of these observations.

## Figures and Tables

**Table 1:** Demographic characteristics of the patients, myeloma isotypes, myeloma staging, and baseline laboratory information.

Characteristics	Number (%)
Total number of patients	47
Age, mean (range)	67 (51–78)
Gender	
Male	29 (61.7%)
Female	18 (38.3%)
Myeloma isotype	
IgG	25 (53.2%)
IgA	11 (23.4%)
Light chain multiple myeloma (LCMM)	7 (14.9%)
Non-secretory multiple myeloma (NSMM)	4 (8.5)
International Staging System (ISS)	
I	11 (23.5%)
II	13 (27.6%)
III	23 (48.9%)
Laboratory values	Median (range)
Creatinine (mg/dL)	2.1 (0.9–4.7)
Urea (mg/dL)	74.1 (33–275)
Calcium (mg/dL)	8.8 (7.4–10.5)
Albumin (g/dL)	2.9 (0.5–3.8)
Hemoglobin (g/dL)	8.2 (6.2–11.4)
Β_2_Microglobulin (mg/L)	7.2 (3–19)
ESR (mm/hr)	95.2 (35–150)
CRP (mg/L)	35.4 (0.3–65)

**Table 2:** Number and percentage of NDMM patients with pathogens.

Pathogen	Number of Patients (%)
Virus	9 (19.1%)
Epstein–Barr virus	4
Herpes Zoster	3
Influenza	2
Bacteria	6 (12.7%)
*Streptococcus pneumonia*	3
*Mycobacterium tuberculosis*	1
*Staphylococcus aureus*	1
*Escherichia coli*	1

## Data Availability

The original contributions presented in this study are included in the article. Further inquiries can be directed to the corresponding author.
